# Innovative BWM–TOPSIS-based approach to determine the optimum delivery method for offshore projects

**DOI:** 10.1038/s41598-025-95710-7

**Published:** 2025-04-17

**Authors:** Lamisse Raed, Ibrahim Mahdi, Hassan Mohamed Hassan Ibrahim, Ehab Rashad Tolba, Ahmed M. Ebid

**Affiliations:** 1 Structural Engineering Department, Faculty of Engineering, Port Said University, Port Fuad, Egypt; 2 Structural Engineering and Construction Management Department, Faculty of Engineering and Technology, Future University in Egypt, New Cairo, Egypt

**Keywords:** Project delivery method (PDM), Offshore project, Risk assessment, BWM, TOPSIS, Civil engineering, Mechanical engineering

## Abstract

Offshore projects hold significant importance in the construction industry by fostering innovation, enabling large-scale infrastructure development, and supporting the expansion of renewable energy sources, enhancing global energy security and economic stability. Effective risk management is crucial in offshore projects to ensure operational safety, sustainability, and financial viability by identifying, assessing, and mitigating potential hazards. Selecting an appropriate project delivery method (PDM) is pivotal for efficient risk management, as it facilitates the proper allocation and mitigation of risks throughout the construction process. This study aims to investigate the impact of PDM on the risk assessment of the lifecycle of offshore platform projects and to identify and evaluate risks associated with offshore projects to improve understanding and optimize performance outcomes. In order To achieve the study’s objective, the Best Worst Method (BWM) and the Technique for Order Preference by Similarity to Ideal Solution (TOPSIS) are utilized for a lifecycle-focused risk assessment to identify the optimum PDM for offshore projects. A BWM–TOPSIS system is developed specifically for offshore projects, starting with organizing risks identified from the literature into a Risk Breakdown Structure (RBS) and subsequent evaluation using the Delphi technique for comprehensive and reliable risk analysis. The findings indicate that Integrated Project Delivery (IPD) and Construction Manager at Risk (CMAR) are the most effective methods due to their higher levels of integration, collaboration, and proactive risk management.

## Introduction

Offshore projects are frequently exposed to various risks; however, these challenges are mitigated by implementing risk management tools and techniques to ensure the safety of employees and organizations^1^. Oil, a critical energy resource for modern economies, is experiencing increasing demand as countries develop and refine their industrial capabilities. The global Offshore Oil and Gas market, valued at USD 119,755.31 million in 2022, is projected to grow at a CAGR of 8.5%, reaching USD 195,358.52 million by 2031, highlighting the increasing reliance on offshore production to meet energy demands. As per the International Energy Agency report in 2023, emerging economies such as China and India are expected to significantly increase their oil consumption in the future^2^.

In 2021, the leading oil and gas-producing nations represented 69.67% of worldwide output. The United States led crude oil and natural gas production, accounting for 26.10% and 34.23%, respectively, among leading producers, with a notable emphasis on unconventional resources such as shale oil and gas. Russia emerged as a leading producer of natural gas and a significant contributor to crude oil production, propelled by its onshore conventional resources. Saudi Arabia excelled in both onshore and offshore conventional crude oil production, making a substantial contribution to world oil supply. Canada achieved a high ranking because to its output of unconventional crude oil, encompassing oil sands and shale gas. Iran and Qatar were distinguished for their offshore natural gas extraction, with Qatar’s North Gas Field serving a pivotal function. The UAE, Iraq, and Norway made substantial contributions, with the UAE concentrating on both offshore and onshore crude oil, Iraq on onshore conventional crude oil, and Norway as a prominent European producer, mostly from offshore areas. These nations collectively underscore the varied sources and tactics in worldwide oil and gas production, encompassing both conventional and unconventional methods^3^. Despite sanctions and limitations, Iran remains a key producer, while Brazil’s offshore resources enhance its production. Due to its significant reserves, Kuwait is a major exporter in the Persian Gulf. These countries’ strategic roles and resources substantially impact global energy policies.

As the world is heading toward more sustainable projects, an effective risk assessment implementation is vital for maintaining sustainability in offshore projects. An effective risk management process plays a crucial role in avoiding delays, cost overruns, and environmental effects, which can impede the long-term capability of offshore operations. Incorporating sustainability objectives into the risk management process helps offshore projects align with environmentally friendly practices, such as decreasing the environmental impact and complying with environmental regulations.

It is essential to integrate environmental standards across the whole project lifespan to guarantee the sustainable execution of the construction process. This process includes the selection of environmentally sustainable suppliers, the implementation of energy-efficient technology, and the minimization of waste. Sustainable construction relies on effective decision-making frameworks that account for uncertainty in supplier performance, resource availability, and environmental effects^4^. Abbasnejad et al.^5^ established a human-technology cooperative framework to achieve significant efficiency targets for the sustainable construction industry. This framework facilitates communication during project realization, shortens construction schedules, increases material efficiency, reduces material and emissions pollution, and enables predictive maintenance evaluation within the project. They stated that the main challenges impeding their broad implementation are high costs, technical barriers, and data management issues.

The project delivery strategy aims to reduce inefficiencies by selecting the most appropriate organizational structure and considering the extent of client participation and risk sharing. Selecting an appropriate project delivery mechanism (PDM) affects the project’s outcome. The three conventional methods are Design-Build, Design-Bid-Build, and Construction Management, each with distinct advantages and disadvantages suitable for various construction projects under different conditions^6^.

**Fig. 1:**
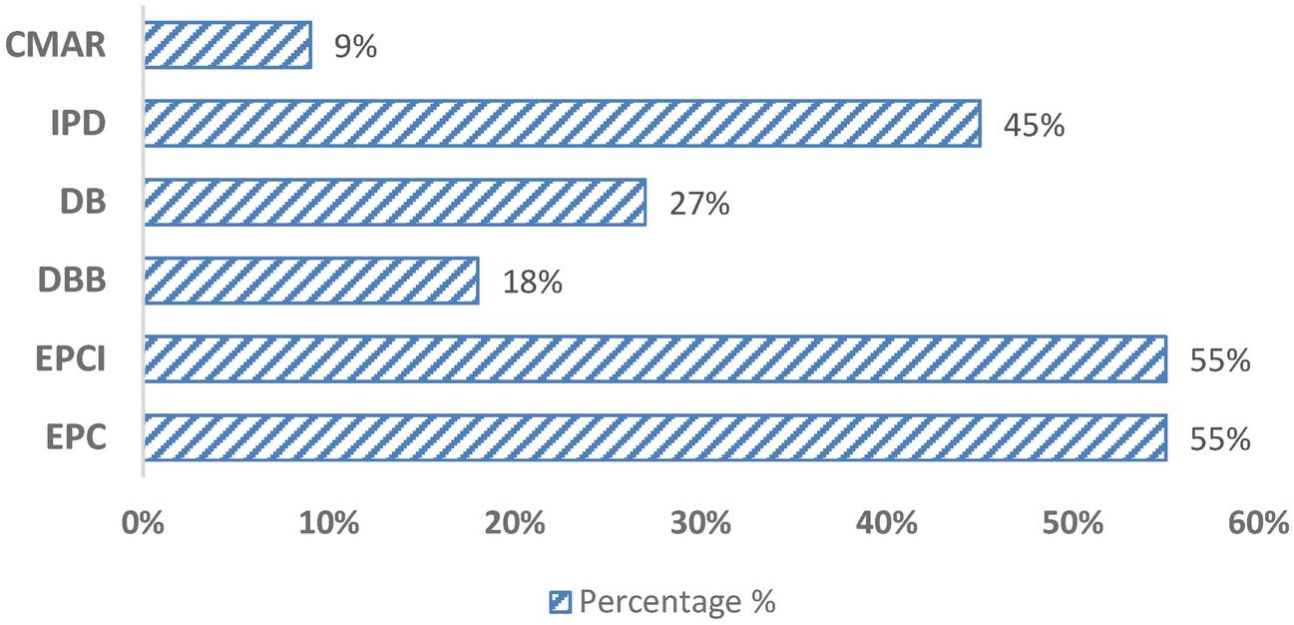
Percentage usage of PDM worldwide.

By exploring databases such as *NS Energy Business*^7^, *Offshore Technology*^8^, *Equinor Energy*^9^, *Gulf Oil and Gas*^10^, and *Aramco*^11^ a comprehensive understanding of global oil and gas projects can be achieved, with insights into project scopes, geographies, and technologies. Approximately 55% of these nations utilize EPC (Engineering, Procurement, and Construction) and EPCI (Engineering, Procurement, Construction, and Installation) as the most prevalent project delivery methods. IPD (Integrated Project Delivery) has gained recent popularity in developed countries such as the USA, China, UAE, Kuwait, and Norway, while CMAR (Construction Manager at Risk) is the least used method employed explicitly in Kuwait. See Fig. 1.

The project management process in oil and gas development typically progresses through a series of critical stages: (1) conceptual design, (2) Front End Engineering Design (FEED), (3) procurement of long-lead equipment, (4) detailed design, (5) construction and fabrication, (6) onshore pre-commissioning, (7) transportation and installation specific to offshore platforms, and (8) hook-up and commissioning before the project is handed over to the end user. The final phase, Operations and Maintenance, focuses on regular maintenance and supervision to ensure the continued functionality and safety of the offshore facility. Each stage is vital and requires precise collaboration across different teams to address the unique challenges encountered in marine environments^12^.

Risk management, a critical aspect of project management, aims to enhance the likelihood of project success. Project risk management involves two main processes: risk identification and risk assessment. Depending on their probability and impact, risks can affect project objectives, including cost, time, scope, and quality^13^. The lifecycle of offshore projects inevitably involves risks. Several studies have highlighted the risks associated with traditional delivery methods in construction projects. However, there is a gap in the research focused on identifying risk factors linked to different project delivery methods throughout the offshore project lifecycle and how these risks influence the choice of the most suitable method. Effective risk management ensures that most project issues are identified early without exceeding the project budget or deviating from the project schedule^14^. Even though extensive studies on risk management in offshore projects exist, significant gaps still need to be studied to understand the impact of various Project Delivery Methods (PDMs) on risk management across the projects’ lifecycle. Additionally, a sufficient decision-making approach for determining the most suitable PDM is lacking. This research gap highlights the need for a systematic approach that combines risk assessment across the project lifecycle with PDM to improve project performance and reduce risks associated with offshore construction projects^15^.

Implementing various Multi-Criteria Decision-Making (MCDM) techniques in construction project management is considered essential due to the complex nature of these projects. Several objectives, such as cost, time management, HSE, and technical performance, are usually encountered in construction projects. Risks across the projects’ lifecycle phases must be considered. These techniques offer a systematic process to evaluate several project factors to ensure that choices consider a complete perspective of the project’s risk profile. They also help project managers consider various projects’ goals in the decision-making process. Furthermore, using MCDM methodologies improves the efficiency of risk management processes. Utilizing various MCDM approaches ensures that risk management in building projects is systematic, data-informed, and effective at dealing with the complex issues associated with large-scale, different activities.

MCDM approaches have been adopted over the years to solve some of the complex project challenges in the construction sector, such as risk management, supplier selection, safety evaluation, and sustainability. It involves making decisions based on numerous criteria that frequently present inconsistencies. Each criterion may exhibit unique characteristics, units of measurement, and relative weight scales^16^. You et al.^17^, utilized a hybrid BWM/TOPSIS methodology to analyze the operational performance of grid organizations, employing BWM for indicator weight calculation and TOPSIS for performance ranking. Furthermore, many research studies have combined fuzzy methodologies with MCDM techniques to address uncertainties and subjective evaluations more effectively, enhancing their adaptability to dynamic conditions. These approaches facilitate the prioritization of alternatives, optimize resource allocation, and increase overall project outcomes by systematically evaluating many criteria. The reviewed studies are summarized in Table 1, which outlines the aims, fields of applications, MCDM used, and how each MCDM is used.

**Table 1:** Overview of MCDM-based research applications in construction and related fields.

No.	Authors and year of publication	Research title	Field of application	Research objectives	MCDM techniques used	How each MCDM was used
1	Mahmoudi et al. 2020^18^	A Novel Model for Risk Management of Outsourced Construction Projects Using Decision-Making Methods	Risk Management in Construction Projects	Develop a decision-making framework to select contracts based on risk management for outsourced construction projects	Best–Worst Method (BWM). Grey Relation Analysis (GRA)	BWM was used to prioritize risk criteria, and GRA was used to rank the contract alternatives based on the calculated weights
2	Yazdani et al. 2020^19^	A Risk-Based Integrated Decision-Making Model for Green Supplier Selection: A Case Study of a Construction Company in Spain	Green Supplier Selection in Construction Projects	Create a structured model to evaluate green suppliers based on risk factors	DEMATEL FMEA, EDAS	DEMATEL analyzed relationships between risks, FMEA evaluated risks associated with suppliers, and EDAS ranked suppliers based on performance
3	Kishore et al. 2020^20^	Designing a Framework for Subcontractor’s Selection in Construction Projects Using an MCDM Model	Subcontractor Selection in Construction Projects	Develop a framework for evaluating subcontractors beyond bid price	Analytic Hierarchy Process (AHP). Simple Additive Weighting (SAW)	AHP weighed criteria, and SAW ranked subcontractors based on normalized values
4	Chen et al. 2019^21^	A Study for Project Risk Management Using an Advanced MCDM-Based DEMATEL-ANP Approach	Project Risk Management	Explore the relationships between project risks, management, and organizational performance	DEMATEL, ANP	DEMATEL analyzed causal relationships, and ANP calculated interdependent criteria weights
5	Tabatabace et al. (2021)^22^	Towards the Success of Building Information Modelling Implementation: A Fuzzy-Based MCDM Risk Assessment Tool	BIM Implementation in Construction Projects	Develop a risk assessment tool for BIM implementation	Fuzzy Delphi, DEMATEL P. FANP	Fuzzy Delphi refined risk factors, DEMATEL analyzed causal relationships, and P-FANP calculated risk weights
6	Mahdi et al. 2021^23^	Decision model to identify the optimum retaining wall type for restricted highway projects sites	Retaining Systems in Construction	Determine the optimal strategy for retaining systems	Analytic Hierarchy Process (AHP)	AHP was used to evaluate different strategies and select the optimal retaining system
7	Mahdi et al. 2019^24^	Decision support system for optimum soft clay improvement technique for highway construction projects	Retaining Systems in Construction	Determine the optimal strategy for soft clay improvements for high way techniques	Analytic Hierarchy Process (AHP)	AHP was used to evaluate different strategies and select the optimal retaining system
8	Elhegazy et al. 2020^25^	Implementing QFD in decision making for selecting the optimal structural system for buildings	Structural System for Buildings	Find the optimal structural system for buildings	Quality Function Deployment (QFD)	QFD was used to analyze and select the best structural system based on predefined criteria
9	Ors et al. 2023^26^	Decision support system to select the optimum construction techniques for bridge piers	Bridge Construction Techniques	Determine the best construction technique for bridge piers	TOPSIS	TOPSIS was applied to rank construction techniques and select the most efficient one for bridge piers
10	Karamoozian et al. 2023^4^	Green supplier selection in the construction industry using a novel fuzzy decision- making approach	Environmentally Sustainable Supplier Selection	Address uncertainty in selecting sustainable suppliers for construction	Best–Worst Method (BWM), PDHFS, SIR	BWM was used to assign criterion weights, and SIR, with probabilistic dual hesitant fuzzy sets (PDHFS), improved the decision-making process for sustainable supplier selection
11	Karamoozian et al. 2023^4^	Risk assessment of occupational safety in construction projects using uncertain information	Occupational Safety Risk Assessment in Construction	Address uncertainties in construction safety risk assessment	DEMATEL Choquet Integral, TOPSIS, IFWA	DEMATEL analyzed risk interdependencies, Choquet Integral was used for analysis, and TOPSIS with IFWA prioritized risks with sensitivity analysis to ensure robust outcomes
12	Karamoozian et al. 2023^4^	Risk assessment of renewable energy projects using a novel hybrid fuzzy approach	Renewable Energy Investment Projects	Identify and prioritize risks in renewable energy investments	IT2HFS, DANP, QUALIFLEX	Interval Type 2 Hesitant Fuzzy Sets (IT2HFS) and DEMATEL-based Analytic Network Processing (DANP) with QUALIFLEX were used to prioritize risks like investment cost and technological maturity
13	Karamoozian and Wu 2024^27^	A Hybrid Approach for the Supply Chain Risk Assessment of the Construction Industry During the COVID-19 Pandemic	Supply Chain Risks during COVID-19 in Construction	Evaluate interrelated supply chain risks linked to the COVID-19 pandemic	Delphi. DEMATEL, IF- TOPSIS	Delphi was used to identify critical risk factors, DEMATEL analyzed risk interdependencies, and Intuitionistic Fuzzy TOPSIS (IF-TOPSIS) ranked the risks based on severity and impact
14	Gidiagba et al. 2023^28^	Sustainable Supplier Selection in the Oil and Gas Industry	Oil and Gas Industry	Develop an integrated MCDM model for sustainable supplier selection in the oil and gas industry	Delphi (criteria filtering). BWM (reduce inconsistencies), TOPSIS (supplier ranking)	Delphi filtered criteria, BWM reduced inconsistencies in weighting, and TOPSIS ranked the suppliers based on the final weights
15	Martins et al. 2020^29^	A Review of the Multicriteria Decision Analysis Applied to Oil and Gas Decommissioning Problems	Oil and Gas Industry (Decommissioning)	Review of MCDM applied to decommissioning in the oil and gas sector	Various Multi Criteria Decision Analysis (MCDA) methods including AHP and DEMATEL	AHP identified important criteria, DEMATEL analyzed the interrelations between the risks
16	Khalidov et al. 2023^30^	Models for the Multicriteria Selection of Options for Decommissioning Projects for Offshore Oil and Gas Structures	Offshore Oil and Gas	Optimize decommissioning processes for oil and gas facilities	AHP. multi-criteria risk assessment	AHP was used to weigh decommissioning options, while multi-criteria risk assessment was used to evaluate quantitative and qualitative risks
17	Wang et al. 2018^3^	A Multi-Criteria Decision-Making Approach Using Hybrid SCOR Metrics, AHP, and TOPSIS for Supplier Evaluation and Selection in the Gas and Oil Industry	Gas and Oil Industry (Supplier Evaluation)	Propose a hybrid MCDM for supplier evaluation in the gas and oil industry	Supply Chain Operations Reference (SCOR) for metrics (criteria), AHP (factor weighting), TOPSIS (ranking)	SCOR was used to select criteria, AHP was used to weigh the factors, and TOPSIS ranked the suppliers
18	Dehdasht et al. 2017^31^	DEMATEL-ANP Risk Assessment in Oil and Gas Construction Projects	Oil and Gas Construction Projects	Propose a risk assessment framework for oil and gas construction projects	DEMATEL (causal relationships). ANP (weighting)	DEMATEL was used to identify causal relationships among risks, and ANP calculated the risk weights based on interdependencies
19	Tsakalerou et al. 2022^32^	An Intelligent Methodology for the Use of Multi-Criteria Decision Analysis in Impact Assessment: The Case of Real-World Offshore Construction	Offshore Construction	Develop a methodology for impact assessment in offshore construction	Various Multi Criteria Decision Analysis (MCDA ) tools	Various MCDA tools were used to evaluate and prioritize stakeholder concerns and project impacts

Selecting the best alternative based on specific criteria is a crucial aspect of construction management. Multi-Criteria Decision Making (MCDM) refers to evaluating various alternatives. Previous studies have utilized several analytical methodologies to support the selection of specific alternatives, such as the Analytic Hierarchy Process (AHP), for determining the optimal strategy and retaining systems^24,23,33^. Quality Function Deployment (QFD) has also been used to find the optimal structural system for buildings^25^. Additionally, the Technique for Order Preference by Similarity to Ideal Solution (TOPSIS) has been used to determine the best construction technique for bridge piers^26^.

By comparing various MCDM techniques, such as AHP, ELECTRE, TOPSIS, PROMETHEE, and VIKOR, Nestico, and Somma observed that TOPSIS efficiently handles a large number of criteria and alternatives while eliminating those with low normalized weights across most features. TOPSIS ranks options according to their closeness to the ideal solution to determine the best alternative across all parameters^34^. MCDM methods, including AHP, TOPSIS (Technique for Order Preference by Similarity to Ideal Solution), and DEMATEL, are particularly helpful in offshore projects, which involve numerous stakeholders and capital investments and are situated in dynamic environments influenced by factors such as weather conditions and regulatory restrictions.

Traditional methods for calculating risk weights have many deficiencies, including inconsistency, subjectivity, complexity, and oversimplification. The Analytic Hierarchy Process (AHP) is comprehensive but can result in inconsistencies when evaluating several criteria. The SAW technique often yields biased and subjective weights. Fuzzy Logic, while useful for expressing uncertainty, employs complex mathematical formulas that impede comprehension. Rezaei presented the Best Worst Method (BWM), demonstrating that BWM requires less comparative data while producing more reliable and precise outcomes^35^. BWM reduces these challenges by minimizing the required comparisons, focusing mainly on high values—the best and worst criteria—thereby decreasing subjectivity while offering a more objective and reliable technique for computing risk weights. Thus, BWM is particularly proficient in delivering an accurate, reliable, and practical risk assessment relevant to complex undertakings like offshore building projects.

An analysis of the strengths and weaknesses of MCDM approaches revealed that the integration of BWM and TOPSIS provides significant benefits for decision-making processes. The Best–Worst Method (BWM) minimizes subjectivity and inconsistency in criteria weighting, whereas the Technique for Order of Preference by Similarity to Ideal Solution (TOPSIS) methodically evaluates alternatives according to their closeness to an ideal solution. The study requires accurate criterion weighting and an effective ranking methodology, enabling the combination of BWM and TOPSIS very effective. This combination guarantees consistent criterion weighting and appropriate ranking of options^36^.

This research utilizes a hybrid BWM–TOPSIS methodology to evaluate lifecycle risks in offshore projects and determine the optimal project delivery mode. In the lack of a systematic database assessing hazards throughout the offshore project lifetime, the Delphi method was employed to obtain expert opinions, hence assuring reliability of data and consistency. This study examines the significance of risk assessment in determining the optimal project delivery method at the beginning phase of offshore projects, employing a hybrid BWM–TOPSIS model to fulfill its aims^37^.

The BWM–TOPSIS model systematically mitigates risks throughout the project lifecycle by assigning weights to essential risks and rating project delivery methods according to their proximity to optimal risk scenarios. A three-tiered technique was established to enhance this model, concentrating on the identification of critical risks within the offshore project lifecycle, prioritizing these risks, and determining the most suitable project delivery method based on the evaluation. This framework offers a methodical and effective strategy for decision-making in offshore initiatives^38^.

## Literature review

### Project delivery methods

Selecting an appropriate project delivery method (PDM) is a complex decision-making process that should occur during the project-scoping phase and certainly before the commencement of the final design process. However, project design often lacks precise and unambiguous specifications^39,40^. Project delivery approaches aim to reduce inefficiencies by assigning the most suitable organizational structure, considering varying levels of client involvement and risk distribution. A PDM, called a procurement route, outlines the organizational framework construction clients use to manage design, construction, and maintenance operations to achieve their goals and objectives by developing completed structures^41^.

Research conducted within the Trinidad construction sector aimed to identify the most suitable PDM for application in a developing context. The study highlighted the significance of the financing cycle and regulatory and legal constraints, emphasizing their influence on the political and regulatory environment in which the delivery method must be implemented^42^.

### Design bid build

The design-bid-build (DBB) approach is the predominant method for global construction services. Typically, the selection of construction firms using this method is based on low-bid procurement^43^. The core principle of DBB involves the client entering into two separate contracts: one with the designer and another with the contractor^44^. In this arrangement, the contractor is primarily responsible for constructing the project according to the design specifications, while the designer’s role is to develop the project design based on the client’s requirements and to supervise the project as the owner’s representative^41^.

Although DBB offers clarity and control, it also presents particular challenges. The linear nature of this method can lead to extended project completion times, and any design issues or changes required post-bid can result in increased costs and delays. Additionally, the separation between design and construction means that contractors often have limited input into the design, potentially leading to practical challenges during the construction phase^45^.

#### Design-build

In the context of design-build (DB), it is essential to recognize that the contractual entity may not always be fully responsible for the design and construction processes due to the variety of design and build approaches. Nevertheless, this method mitigates the competitive relationship between design and construction teams in traditional methods. As a result, fostering innovation, communication, and cooperation throughout the project lifecycle reduces cost and time while enhancing client satisfaction^46^.

A notable advantage of the design-build approach is its ability to provide the owner with a single point of contact through a main contractor. However, this process is more complex than it might initially seem. Different iterations of the design and build approach exhibit unique characteristics that deviate from the precise definition provided by Crossman^47^.

**Fig. 2:**
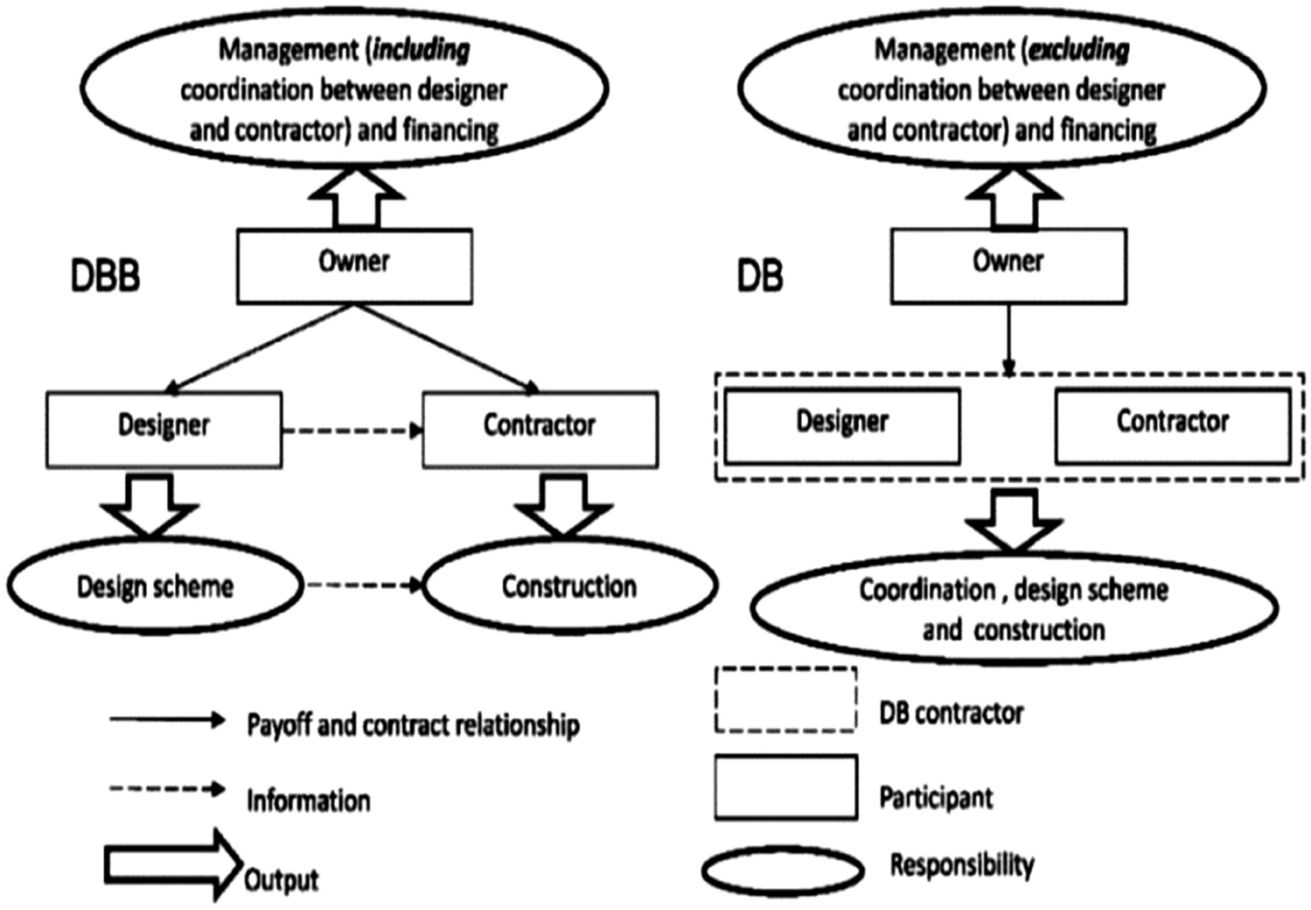
Difference between DBB &DB^48^.

The distinction between DB and design-bid-build (DBB) delivery approaches lies in the contracting strategy. In Design-Build approach, the design and construction services are combined into a single contract with one entity. Conversely, the design-bid-build separates these services into two contracts, with the design phase completed before the construction phase is put out for bidding and awarded to a contractor, as illustrated in Fig. 2^48^.

#### Construction management at risk

The Construction Manager at Risk (CMAR) project delivery method has recently gained popularity, particularly within the public sector^6^. CMAR was identified as the most suitable project delivery strategy for infrastructure projects due to its unique integration of the owner’s project control and the agency manager’s expertise, both critical factors influencing the project’s overall success. This balanced approach fosters a collaborative relationship between the owner and the manager, enhancing project management by facilitating comprehensive project control, efficient risk allocation, cost reduction, shortened project timelines, minimized need for specialized skills in the owner’s project team, and ensuring long-term project sustainability. A significant attribute of CMAR is the early involvement of the construction manager, promoting a collaborative approach to project planning and design. This early engagement allows the construction manager to provide valuable input on cost estimation, materials selection, and constructability issues, leading to more accurate budgeting and scheduling^49^.

#### Public private partnership (PPP)

Public–private partnership (PPP) is a highly significant approach to project delivery. Developed countries have employed this method to execute infrastructure projects across various sectors, such as transportation, telecommunications, power, water, sanitation, health, education, and correctional facilities, aiming to ensure project sustainability^50^. Following the contract award and financial closure, PPP projects advance into the partnership phase (P3), encompassing project construction, operation, and maintenance. The partnership phase is a critical component of the PPP lifecycle, as it marks the commencement of the implementation of a PPP project^51^. Infrastructure projects under PPP arrangements are particularly vulnerable to numerous risks, including technical, construction, operations, revenue, financial, resources, production, force majeure, political, regulatory, environmental, commercial, and unforeseen hazards^52^.

#### Build-operate-transfer (BOT)

The Build-Operate-Transfer (BOT) model is widely used for delivering infrastructure projects, particularly in developing countries^53^. In BOT projects, the private partner is responsible for constructing a facility that meets the agreed-upon criteria set by the public entity. After construction, the private partner operates the facility for a specified period before transferring ownership to the public entity at the end of the concession term. The project is designed to generate sufficient returns to cover the private sector’s investment over the concession period^54^. Investors must adopt several critical success factors (CSFs) to consistently secure BOT contracts. Key elements agreed upon by both parties typically include selecting the right project, leveraging the consortium’s strengths, offering a superior technical solution, presenting a unique financial package, providing guarantees, and demonstrating strong entrepreneurship and leadership. Integrating and prioritizing these factors can significantly enhance the likelihood of winning a BOT contract^55^.

#### Integrated project delivery (IPD)

Integrated Project Delivery (IPD) is a project implementation strategy designed to enhance contractual arrangements, improve precision in planning, boost productivity through collaboration, and increase communication effectiveness, thereby addressing the construction industry’s lag relative to other sectors^56^. The use of appropriate technology is a critical component in the implementation of IPD^57^. Adopting these concepts eliminates traditional contractual boundaries, leading to a clearer delineation of objectives and duties^58^.

Key contractual elements of IPD include equal rights for all parties, shared risks and profits, equal accountability, and early participation from all involved stakeholders. Behavioral components encompass mutual respect and trust, willingness to collaborate, open communication, collaborative definition of project objectives, and collective decision-making^59^. For contractors, essential aspects include cost reduction, precise planning, and profit sharing^60^. Shared goals between the employer and contractor, such as minimizing waste, claims, and resource inefficiencies, are crucial^61^.

IPD faces various challenges, including organizational, environmental, and capital macro factors. Financial difficulties represent a capital challenge, while organizational issues include managerial, contractual, educational, communication, and technological problems. Environmental concerns encompass cultural, legal, and political difficulties^55,62^.

#### Engineering, procurement and construction (EPC)

This approach integrates planning, material procurement, construction, and risk management under a single contract, with the general contractor in EPC projects managing these aspects from the initial phase, including immature technical and social elements ^39,63^. Under this model, driven by advancements in the building industry, the contractor is responsible for ensuring quality, safety, timeliness, and cost control. The complexity of the construction process and various contributing factors, such as the building environment and managerial skills, exacerbate the challenges of completing projects^64^.

**Fig. 3:**
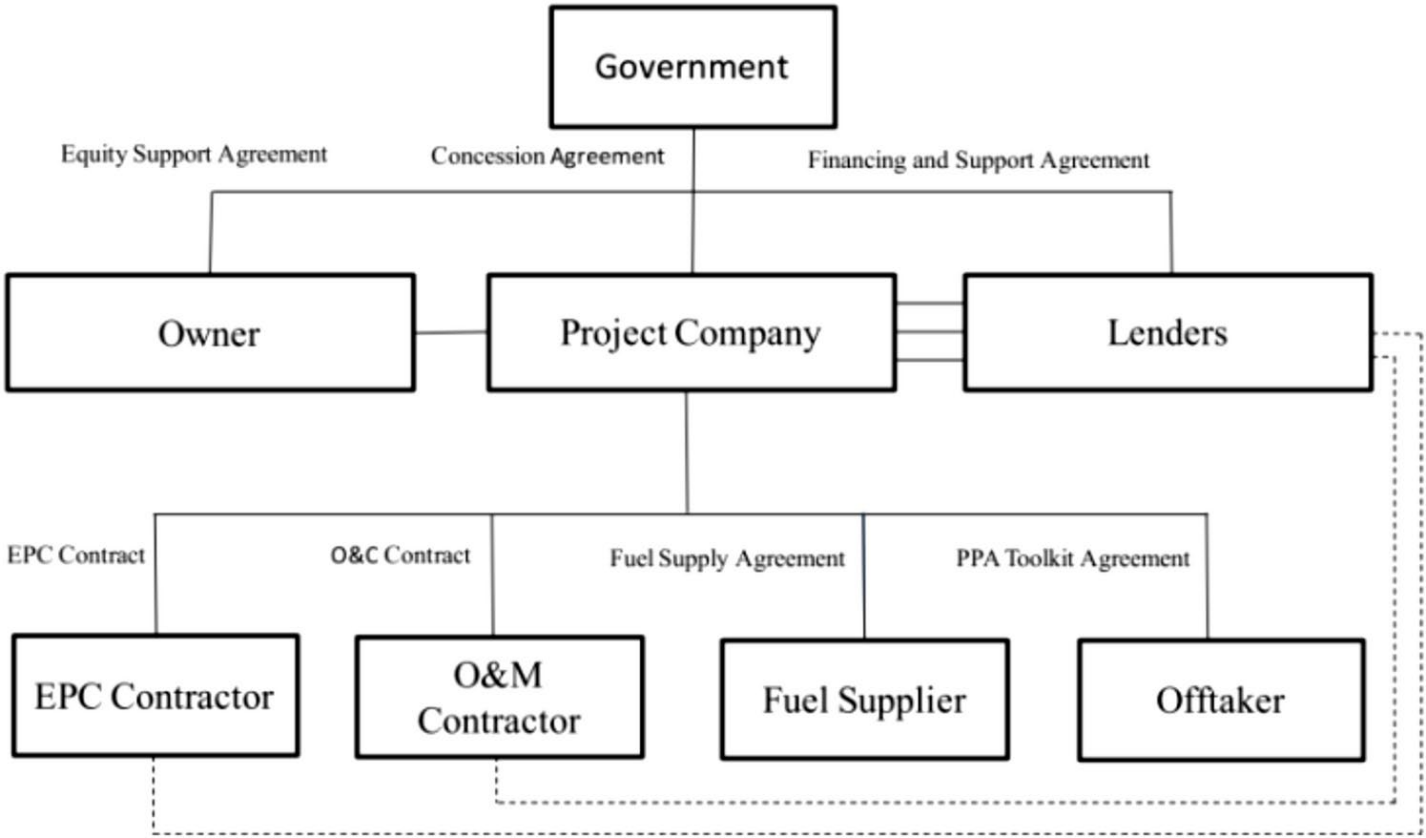
The basic structure of the EPC contract^65^.

EPC contracts typically address these challenges using more sophisticated techniques than traditional construction contracts. Effective implementation of the EPC strategy requires the contractor’s understanding, control, and ability to influence risk distribution and their readiness to accept the associated risks^65^. Figure 3 illustrates the fundamental structure of EPC.

### Risk management

#### Risks in project delivery method

Establishing a comprehensive set of criteria for selecting the appropriate project delivery method (PDM) significantly enhances the clarity of the project’s current status and refines the selection process. Extensive reviews have identified various factors influencing PDM selection, offering insights on how these criteria can be customized to address the specific requirements of diverse projects^66^. Four fundamental principles for effective risk allocation have been proposed, which include assigning risks to the party best equipped to manage them, ensuring that risk allocation aligns with the project’s objectives, sharing risks when they contribute positively to project outcomes, and striving to distribute risks in a manner that promotes team collaboration while aligning with customer-focused performance goals^67^. Additionally, it has been observed that risks within complex environments are often highly interconnected, resulting in amplified impacts^68^. Consequently, project management teams must collectively comprehend the cumulative effects of all project risks rather than formulating strategies for individual risks in isolation.

#### Risk Management in offshore projects

Risk management related to oil and gas construction (OGC) projects has been sporadically studied, with limited dedicated analyses focusing on this sector. Van Thuyet et al.^69^ conducted an in-depth assessment of 59 major risk factors within OGC projects in Vietnam, identifying ten as particularly critical, thereby indicating a growing but insufficient focus on comprehensive risk analysis in this high-stakes field. Building on these foundational studies, Mubin and Mannan^70^ explored the risk factors associated with Engineering, Procurement, and Construction (EPC) contracts, identifying 168 risks and categorizing them into seven groups. Similarly, research by Kassem et al.^71^ identified up to 51 risk factors in the Yemeni oil and gas sector, which were categorized into internal and external risk factors, highlighting the complex nature of risks inherent to this industry. Lenkova^72^ and others provided detailed categorizations of risks, dividing them into internal and external factors with subsequent structuring within each group, which is crucial for systematically managing risks by understanding their origins and potential impacts on projects.

In a comprehensive literature review, Dey et al.^73^ explored the general causes of project failures and risk factors that affect project success in the Indian oil industry, categorizing these risks across different phases of project development, including planning, implementation, and evaluation, while also addressing key areas such as project management processes, organizational transformation, and technology management. Khalilzadeh et al.^74^ focused on identifying and assessing key risks in the oil and gas sector, particularly during times of sanctions and uncertainty, identifying critical risks during the exploration and exploitation phases and categorizing them into seven groups: time and cost, human resources, quality, procurement, scope, communications, and other primary risks. Their study emphasizes the importance of management’s continuous risk identification and evaluation to mitigate impacts or likelihoods, facilitating an understanding of how managing one risk can influence others. However, the study acknowledges that not all risks can be eliminated, especially those with inherent traits that require careful consideration in managerial decision-making. Ketabchi and Ghaeli^75^ employed the Fuzzy Best Worst Method (FBWM) for risk weighting, identifying technical risk and project execution as significant concerns, particularly in offshore projects. This methodological innovation provides a quantitative approach to understanding and prioritizing risks. The necessity for an integrated methodology to effectively manage construction risks is emphasized in research by Al Mhdawi^76^, which focuses on oil and gas fields in Iraq and supports the development of analytical models and structured methodologies to formalize human knowledge and risk characteristics. Kraidi et al.^77^ identified several key risks affecting projects, including inadequate safety regulations, poor inspection and maintenance practices, limited detection and monitoring of potential threats, and a general lack of awareness regarding legal and moral responsibilities. Other significant issues noted include design, construction, and material defects, insufficient training, threats to employee safety, inadequate risk documentation, vulnerable infrastructure such as easily accessible pipelines, insufficient warning systems, a lack of modern IT services and equipment, frequent operational errors, disputes over land ownership, and inadequate research on relevant topics. In the oil and gas industry, implementing an effective risk management process is crucial in several key areas, as outlined by Alnoaimi and Mazzuchi^1^, including conducting job safety analyses to prevent workplace accidents, adhering to government regulations, managing environmental impacts, balancing supply and demand fluctuations, and controlling costs. Oil and gas companies are vulnerable to losses or crises due to any identified risk, making it imperative for these entities to monitor negative events to anticipate potential emerging risks. By analyzing these emergent risk properties, companies can foresee future hazards and identify potential new opportunities for enterprise development, as highlighted by Alnoaimi and Mazzuchi^1^. This proactive approach to risk management aids in navigating both the challenges and opportunities present in the dynamic oil and gas field.

## Research methodology

**Fig. 4:**
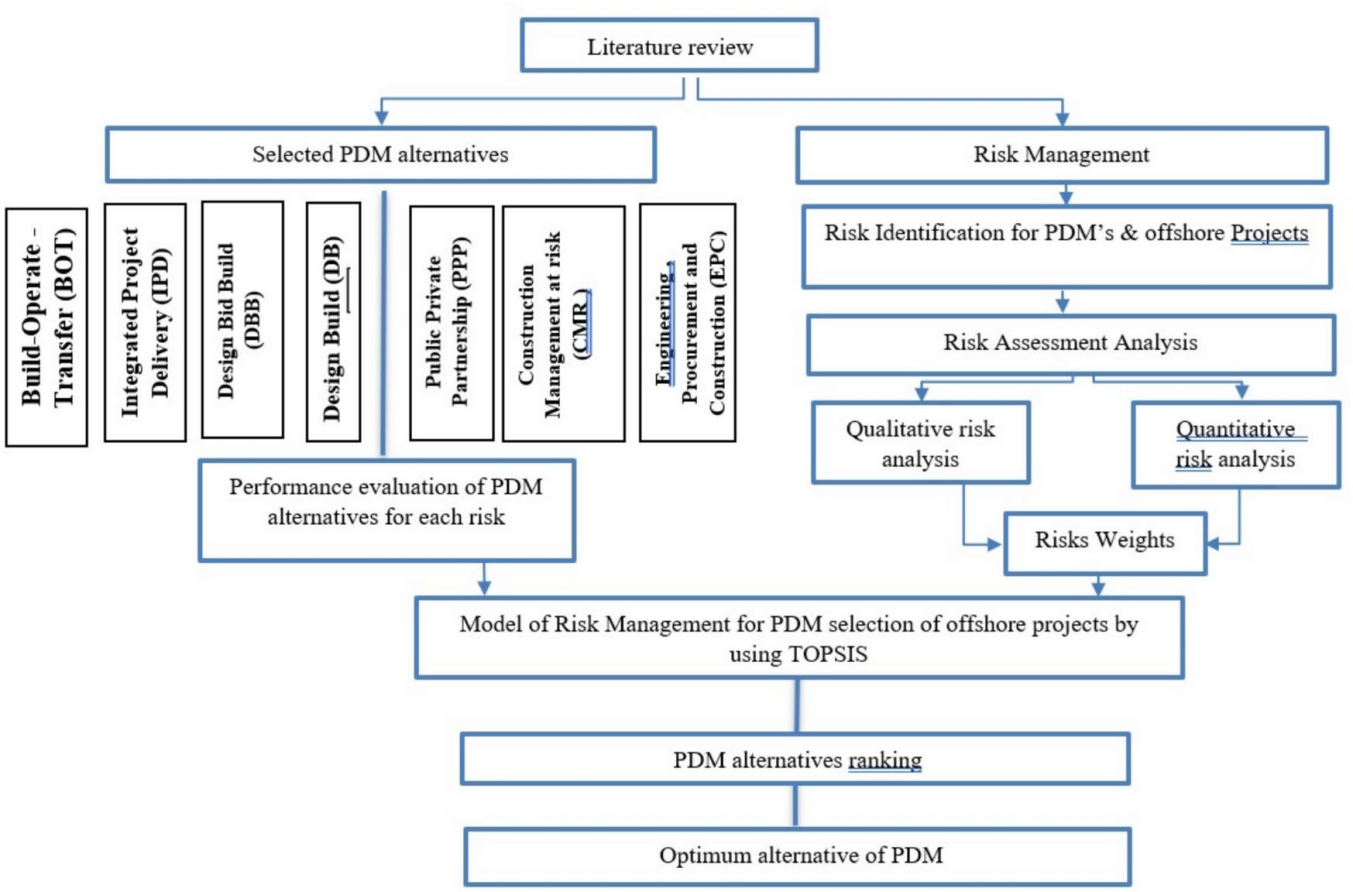
Research methodology.

This research focused on identifying potential risk parameters affecting the lifecycle of offshore projects by reviewing existing literature and resources. Building on established factors, the study aimed to enhance current knowledge and address relevant risks that could influence offshore project outcomes throughout their lifecycle. A closed-format questionnaire was developed as the initial step to systematically recognize the potential risks and challenges associated with offshore projects. This questionnaire gathered data on hazards throughout the lifecycle of offshore projects, which were then organized into a recommended framework. Experts were invited to incorporate, modify, or eliminate elements as necessary, ultimately achieving consensus among them by the conclusion of the process. The initial stage involved compiling fundamental structured data on hazards sources from the literature, resulting in a Risk Breakdown Structure (RBS) tailored explicitly for offshore projects. The questionnaire was designed to obtain expert assessments regarding the probability of risks occurring and their potential impact on project objectives. Figure 4 summarizes the considered methodology.

The distinctive characteristics of offshore projects pose challenges in sourcing a sufficient number of highly skilled workers, which may have affected the diversity of insights collected. Despite the limited sample size, the interviewed experts offered valuable and comprehensive insights crucial for understanding the complexities of offshore projects, drawing from their extensive experience in industry-related risk assessments.

The RBS served as the input for evaluating the probability and impact of the identified risks. Using quantitative research and expert opinions, 27 significant risks were selected for inclusion in the most appropriate project delivery approach selection model. These risks were systematically categorized into four groups for comprehensive analysis. The first category, Management Risks, encompasses issues related to project planning, decision-making processes, and organizational structures that could influence project success. The second category, Financial and Economic Risks, includes factors such as budget overruns, funding challenges, and economic instability that could jeopardize the project’s financial viability. The third category, External and Site Conditions, pertains to environmental factors such as ecological changes, ethical standards, and site-specific issues. Lastly, Technical Risks involve design, software engineering, and technology usage challenges that may result in operational difficulties or failures.

Due to the limited sample size in offshore projects, noticeable variations in risk weights emerged between the two rounds of questionnaires. The initial questionnaire concentrated on a smaller sample size to identify the most critical risks, while a larger sample size was employed in the second round to ascertain more precise risk weights. This approach, which yielded a more accurate and representative assessment of previously highlighted hazards, ensured a thorough and balanced evaluation. The process underscores the significance of sample size in accurately assessing and prioritizing risks in offshore projects.

Risk assessment and prioritization of Project delivery methods (PDM) within the context of offshore construction projects have been suggested in this research through the integration of the following techniques: extensive literature review, Delphi technique, Best–Worst Method (BWM), and Technique for Order Preference by Similarity to Ideal Solution (TOPSIS). Risk exposure is carried out using an extensive literature analysis relating to the two areas of concern: offshore construction projects and various PDMs. It follows a complete approach to risk analysis using the Delphi technique, wherein opinions are obtained from experts, and the trend of risk preference forms the basis for BWM. BWM calculates the risk weights in all the life cycle stages of an offshore project, hence quantifying the risks. Finally, the PDMs were ranked through TOPSIS, which integrates various aspects of risk issues into the decision-making process. The application of modern decision-making tools, including Delphi, BWM, and TOPSIS, in an integrated approach has provided a structured, efficient, and reliable way of performing risk management and optimization related to PDM and opened new evaluation dimensions in an offshore construction project.

The Delphi method typically commences with an initial round of feedback from a smaller, specialized group of experts, usually comprising approximately eight participants. This approach facilitates a comprehensive exploration of key issues and the development of a robust framework for the investigation. As the process advances to the second round, expanding the sample size to around 30 experts is beneficial, allowing for a broader range of perspectives and enhancing the depth of understanding. This increased diversity of expert viewpoints bolsters the reliability and accuracy of the findings, ensuring that the conclusions drawn are robust and representative of the wider expert community. Selected experts met at least one of the following three criteria: a minimum of eight years of experience overseeing offshore projects, ten years of academic research experience in offshore projects, or possessing a master’s degree in petroleum project management.

**Fig. 5:**
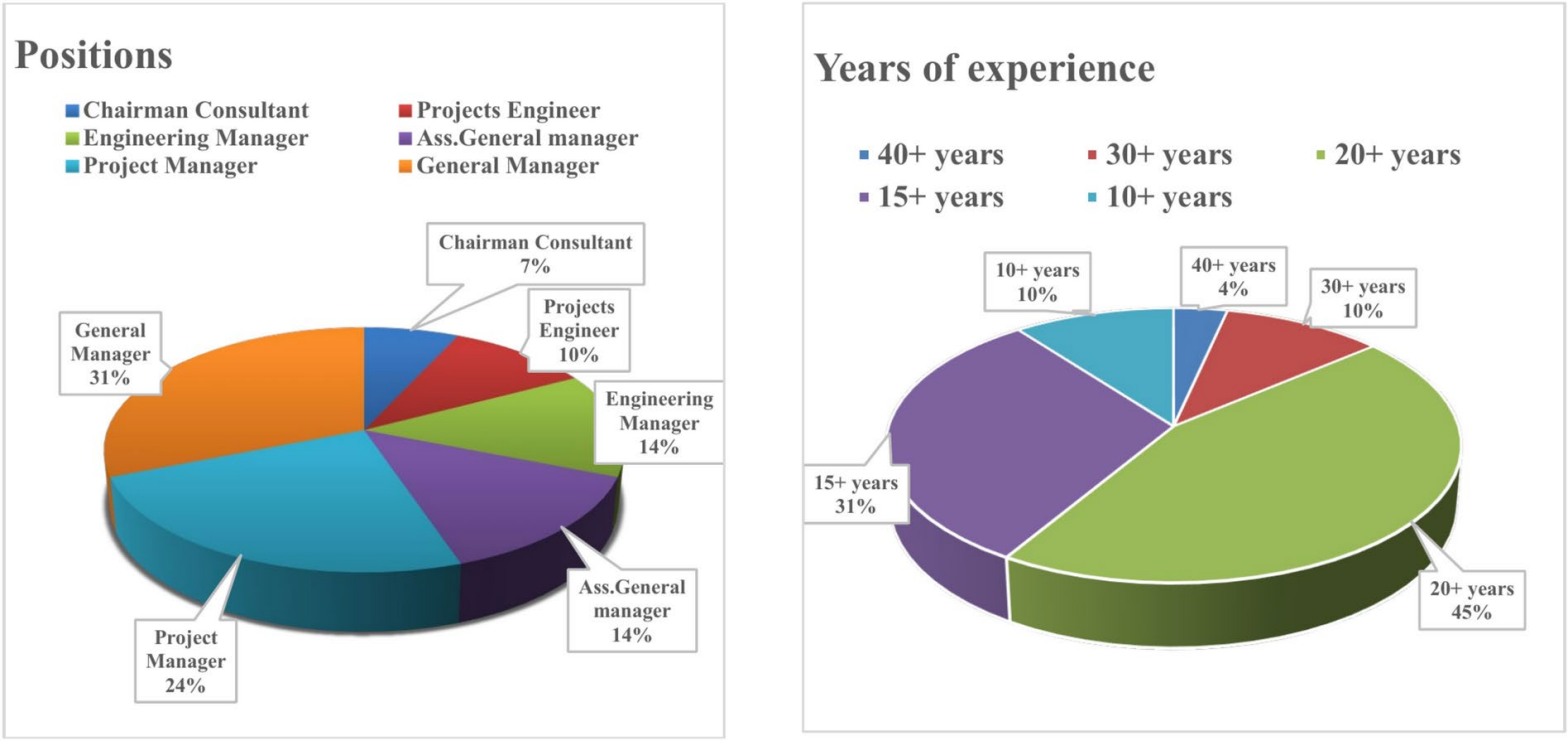
Participants positions and years of experience.

Figure 5 shows an overview of positions and years of experience for surveyed participants. The majority of respondents are General Managers (31%) and Project Managers (24%), followed by Engineering Managers (14%) and Assistant General Managers (14%). Years of Experience chart highlights that 45% of participants have over 20 years of experience, with 31% having more than 15 years. This distribution indicates a strong presence of experts in senior roles.

## Risk identification for offshore projects

### Risk analysis to develop the selection criteria

**Fig. 6:**
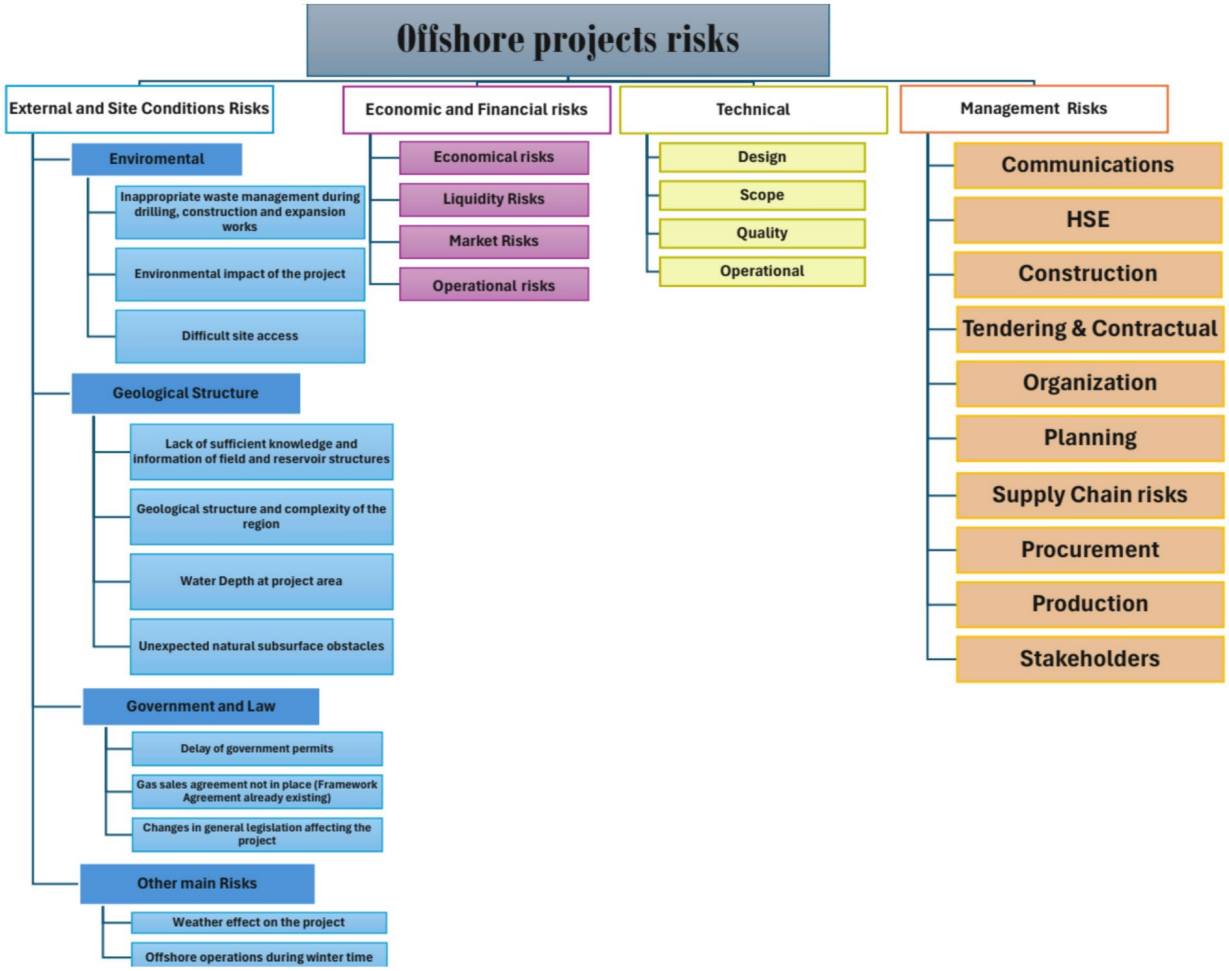
Risk breakdown structure.

Due to the lack of a unified database detailing risks associated with the lifecycle of offshore projects, initial risk identification was undertaken by reviewing relevant literature and examining available technical documentation. This comprehensive review resulted in the identification of 117 distinct risks. Subsequently, a detailed inventory of potential risks for offshore project delivery was compiled, accompanied by a classification scheme for effective organization. This effort culminated in developing a Risk Breakdown Structure (RBS) for offshore drilling projects, which serves as a standardized repository of potential uncertainties. The RBS is organized hierarchically, featuring a top-level category, four primary risk areas, 26 subcategories, and a total of 117 specific risk items. The structured list of risks is delineated in the RBS for offshore projects, and Fig. 6 visually illustrates the four-tiered structure of the risk categories, with particular emphasis on external and site condition risks.

Expert opinions were utilized to evaluate the probability of risks and their potential impact on project objectives. Data collected from interview questionnaires were aggregated, with the total scores for each risk factor divided by five to derive the average, reflecting the overall score from all participants. The relative position of each factor indicated its level of risk performance, providing valuable insights into the risk management objectives of stakeholders. The estimated impacts of risks were inputted into relevant templates, which Primavera Risk Analysis subsequently utilized to construct a risk model, also referred to as an affected risk plan, for the project schedule. Developing this risk model involves estimating the potential consequences of identified risks and applying these to the project’s cost and schedule.

The risk score presents an overall risk effect on project objectives. Based on the 27 high risks that have been identified, the cutting line for top risks was determined to be a minimum score of 20 on project objectives.

Dividing the offshore project lifecycle into three distinct phases—development, Construction, and Operation—provides a structured approach to risk analysis and allows for targeted strategies at each phase.

- *Development Phase* risk analysis focuses on regulatory approvals and financial feasibility, addressing uncertainties that could impact the project’s viability before significant investments are made.
- The emphasis shifts to physical and logistical risks in the **construction phase**, such as engineering challenges, supply chain logistics, and workforce safety. This phase requires rigorous contingency planning to manage potential delays and cost overruns.
- *Operation Phase* involves risks associated with the project’s ongoing maintenance and operational efficiency.

### Results of best worst method (BWM)

The calculation of risk weights derived from the Best–Worst Method (BWM) is based on a structured process following identifying top risks from the first round of the Delphi technique. In the initial stage, data from expert evaluations is gathered, identifying the most critical risks in various categories—such as management, technical, financial, and external risks—based on the offshore project’s life cycle phases (development, construction, and operation). In the second round of the Delphi process, BWM is applied. Experts are asked to select the most important and least important risks within each category for each project phase. Additionally, they are asked to assess the preference of every other risk in relation to the most and least important risks. This preference scoring provides relative comparisons between risks. The data collected from these evaluations are then used to calculate the weights of each risk, ensuring that the most critical risks are given higher weightings. Combining expert judgment with BWM, this structured approach provides a consistent and accurate prioritization of risks across the project’s life cycle.

Table 2 represents the weight of each risk resulting from Best -worst method for project life cycle phases.

**Table 2:** Risks weights (BWM results).

Risk category	Risk title	Weight	Consistency ratio
Development phase			
Technical risks	Design errors	0.60	0.038
	Changes in the scope of the project	0.40	
Financial and economical risks	Changes in Economic policies	0.38	
	Currency Fluctuation	0.36	
	Suppliers Bid greater than estimate	0.26	
Management risks	Improper Project Feasibility Study	0.21	
	Failure to select the type of implementation on the project contract	0.18	
	Improper project planning and budgeting and lack of accurate estimation of project value	0.18	
	Bad identification of equipment and material	0.16	
	Delay of tender offer evaluation and purchase order cycle	0.14	
	Low level of engagement of disciplines in planning across the project	0.12	
Construction phase			
External & site conditions	Lack of sufficient knowledge and information of field and reservoir structures	0.25	0.096
	Delay of government permits	0.24	
	Environmental impact of the project	0.22	
	Weather effect on the project	0.16	
	Water Depth in the project area	0.13	
Financial and economical risks	Project financing availability (debts & delayed payment on contract)	0.31	
	Cost over-run (bad initial cost estimation)	0.28	
	Currency fluctuation (foreign exchange rate)	0.22	
	Change in economic policies	0.19	
Management risks	Bad application of safety	0.25	
	Failure to achieve the main target point	0.22	
	Bad start-up plan	0.19	
	Lack of engineering resource qualifications and pool depth	0.18	
	Delay of engineering designs during work	0.16	
Technical risks	Bad quality control	0.27	
	Changes in the scope of the project	0.2	
	Design errors	0.19	
	Design changes during construction	0.18	
	Construction mistakes	0.16	
Operation phase			
External and site conditions risks	Environmental impact of the project	0.52	0.024
	Weather effect on the project	0.28	
	Water Depth in the project area	0.2	
Management risks	Bad application of safety	0.48	
	Inadequate qualifications of engineering experts	0.28	
Technical risks	Bad quality control	0.42	
	Inappropriate operating methods	0.37	
	Failure to predict the exact time to run the project	0.24	
	Error in forecasting demands for service	0.21	

Financial risks are particularly pronounced during the start-up phases of offshore construction projects due to the substantial initial investments required. These risks are influenced by market volatility, currency value fluctuations, and the costs associated with conducting preliminary research, environmental assessments, and acquiring necessary licenses and permits. At this stage, any inaccuracies in cost estimation or unanticipated expenses could jeopardize the project’s sustainability. Consequently, meticulous risk management and financial planning are critical to ensure project success.

During the development phase, there is an increased vulnerability to design errors. Even minor inaccuracies can lead to significant rework, project delays, and safety concerns. As the project progresses, rectifying these errors may become more challenging and costly, potentially resulting in timeline extensions and budget overruns. Therefore, thorough inspections and precise design reviews are essential to identify and rectify shortcomings early in the process, facilitating smoother project advancement.

The construction phase of offshore projects presents notable risks, particularly concerning financing availability and the potential for rising costs. Financial challenges pose substantial threats to the timely and effective execution of projects. Additionally, delays in securing government approvals may disrupt project schedules and increase costs.

Among the most significant risks identified during the operational phase are the environmental impacts associated with the project. The potential harm to local ecosystems, water quality, and marine life can result in long-term ecological damage and stringent regulatory consequences. Inadequate safety measures may lead to accidents and injuries, adversely affecting team morale and incurring legal penalties. Furthermore, poor quality control represents a significant risk, as it can result in substandard construction, structural deficiencies, higher maintenance costs, and a diminished project lifespan. Employing improper operational techniques can undermine the efficiency and effectiveness of a project, leading to delays, increased costs, and operational hazards. Notably, water depth at the project site is considered a lower-risk factor during both the construction and operational phases, as preliminary studies and historical data indicate adequate water depths in the project locations, thereby reducing the likelihood of hazardous conditions.

### Prioritize project delivery methods alternatives using TOPSIS

The primary objective of this study was to develop a model that facilitates the selection of the optimal project delivery method through comprehensive risk analysis. It examined seven project delivery methods commonly employed in the construction industry: Design Bid Build (DBB), Design-Build (DB), Construction Manager at Risk (CMAR), Public–Private Partnership (PPP), Build-Operate-Transfer (BOT), Engineering, Procurement and Construction (EPC), and Integrated Project Delivery (IPD). A thorough review of the literature was conducted to assess these methods. The study evaluated the influence of risks on project objectives throughout the project lifecycle and employed the Technique for Order Preference by Similarity to Ideal Solution (TOPSIS) to rank these methods based on their risk profiles and overall impact assessments.

By incorporating risk, weights calculated using the Best Worst Method (BWM) into the TOPSIS analysis, the decision-making process for selecting the optimal project delivery method is significantly enhanced. The BWM effectively determines the relative importance of each risk through comparative analysis of the best and worst criteria, thereby establishing a precise and prioritized weighting system. These weights are subsequently utilized within the TOPSIS framework, which ranks the various project delivery options by measuring each method’s proximity to an ideal solution. By using weighted risks as a critical factor in the TOPSIS analysis, decision-makers can ensure that the evaluation of project delivery methods is comprehensive and accurately reflects the significance of each risk concerning the project’s success. This integrative approach offers a robust framework for objectively assessing the potential effectiveness of each delivery method in managing the inherent risks associated with construction projects.

### Ranked PDM

In assessing the efficiency of various project delivery methods utilizing the TOPSIS approach, the ranking of methods is determined by the normalized distance (Dj) values calculated for each method. These (Dj) values indicate the proximity of each method to the ideal solution, which directly influences their respective rankings. The findings illustrate a comparative analysis of different project delivery methods throughout the lifecycle of offshore projects based on their normalized weights. Integrated Project Delivery (IPD) emerged as the most effective method, with a weight of 0.188, indicating a 2.4% higher effectiveness than Construction Manager at Risk (CMAR) and a 7.5% higher effectiveness than Public–Private Partnership (PPP). The IPD approach is distinguished by its superior integration and stakeholder coordination, rendering it particularly suitable for complex offshore projects.

**Fig. 7:**
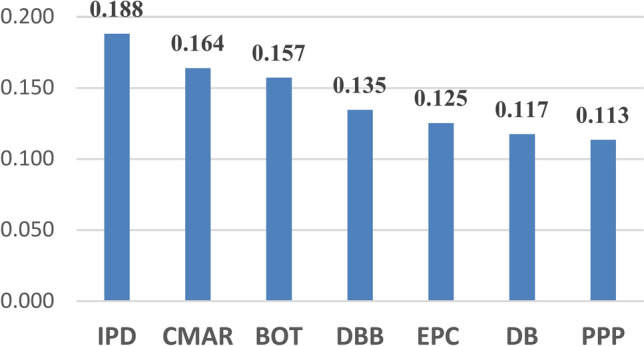
Average normalized weights for all project’s phases.

**Fig. 8:**
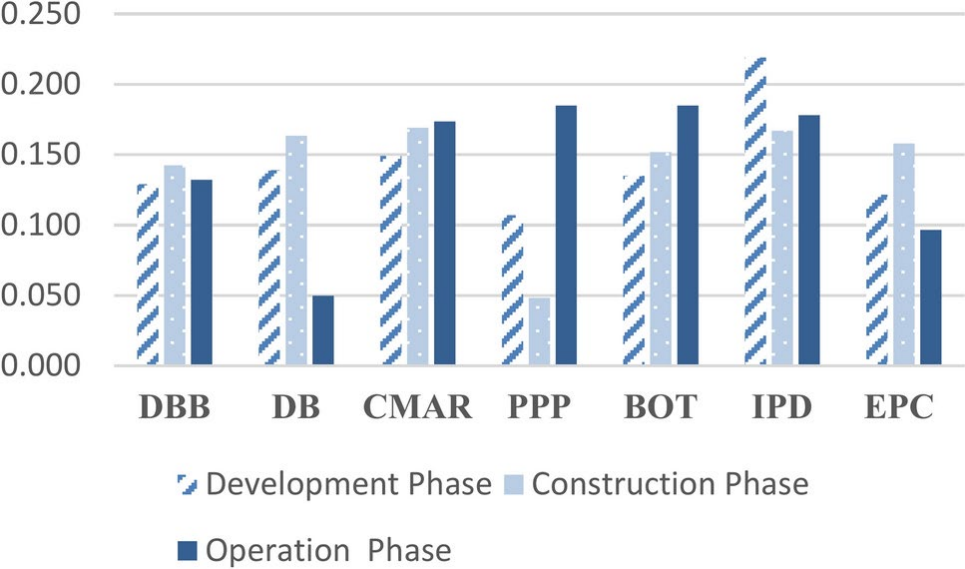
Normalized weights for all project phases.

Meanwhile, CMAR demonstrates high effectiveness due to its emphasis on early risk management. Build-Operate-Transfer (BOT) is advantageous for projects necessitating a long-term operational focus. The Engineering, Procurement, and Construction (EPC) method provides a single point of responsibility, ensuring consistency; however, it relies heavily on the contractor’s expertise, achieving a 1.2% higher effectiveness than PPP. The Design-Build (DB) method may encounter challenges related to diminished monitoring and quality control. The delivery methods ranking are summarized in Table 3 and graphically presented in Figs. 7 and 8.

**Table 3:** Delivery methods ranking.

	Development phase		Construction phase		Operation phase		Overall ranking
	Normalized weight	Rank	Normalized Dj	Rank	Normalized Dj	Rank	
DBB	0.129	5	0.142	6	0.132	5	0.135
DB	0.139	3	0.163	3	0.050	7	0.117
CMAR	0.149	2	0.169	1	0.174	4	0.164
PPP	0.107	7	0.048	7	0.185	1	0.113
BOT	0.135	4	0.152	5	0.185	2	0.157
IPD	0.219	1	0.167	2	0.178	2	0.188
EPC	0.122	6	0.158	4	0.097	6	0.125

The study indicates that IPD is the optimum PDM, followed by CMAR and BOT; hence, it provides insight into the effective management of complex offshore projects. It also brings out the importance of IPD could be due to its nature in ensuring cooperation, sharing of risk, and integrating stakeholders in ways that become of essence in high-risk environments like offshore projects. While a tendency toward CMAR reflects the importance of early contractor involvement and proactive risk management, BOT has shown its potential in large infrastructure projects. There is a high probability that application of the IPD/CMAR methodology will significantly reduce the significant risks high risks weights derived from BWM for risk categories in every phase of an offshore project, including the risks of design inaccuracy, project scope changes, changes in economic policy, changes in currency exchange rate, and project financing problems.

IPD allows integration and collaboration between owners, contractors, engineers, and suppliers from the project’s initiation phase. These findings would be helpful to project managers and decision-makers in the offshore construction industry, as they provide evidence-based guidelines on selecting the best PDM for a project’s complexity, risk profile, and lifecycle concerns.

## Validation

A case study (located in Egypt) was chosen to validate the proposed hybrid BWM–TOPSIS model by investigating a real-world example that supports the study’s objectives. The available real data of the selected case study provided a source of offshore project risk assessment, enhancing the analysis’s credibility.

The model findings with actual project outcomes were compared to evaluate the model’s sensitivity to the different risk scenarios associated with offshore projects. Project conditions, the number and type of identified risks, and the comprehensiveness of the risk assessment process impact ranking changes.

This case study discusses a real offshore project in the Egyptian sector. During the project’s life cycle, including the development, construction, and operation stages, 52 risks were identified and categorized into four main classes.

The Validation process for the optimum PDM selection based on risk analysis started by identifying all the potential risks that could impact PDM selection. These identified risks were then categorized into four main categories: external and site conditions, technical and management risks, and financial and economic risks. Moreover, these risks were categorized as part of the project’s development, construction, and operational phases. After this, the BWM is applied. Experts were asked to select each category’s most important (best) and the least important (worst) risks for all project phases. Concerning their choices, the relative importance of each risk is determined, and the risk weights are calculated by applying the BWM Solver in Excel. Then, for each identified risk, the occurrence probability and the impact level for each PDM are determined. Finally, TOPSIS calculates a normalized weight for each PDM by ranking the potency of all methods to mitigate the identified risks.

The identified risks were grouped as follows: Management risks were the most prominent in 23 instances (44%), highlighting the challenges of project planning, coordination, and leadership involved during the entire course. Technical risks constituted 15 instances (29%) that highlighted engineering and operational challenges experienced, in particular during the construction phase. Accordingly, the Financial & Economical risks included 6 risks, or 12%, concerning budgetary limitations, cost overruns, and the greater economic climate, while it influenced all phases with a greater emphasis on the development and operation stages. The External & Site Conditions risks totaled 8 risks, or 15%, influencing environmental factors, regulatory challenges, and site-specific issues, primarily impacting the construction and operation phases. The distribution of risks along the life cycle shows a significant share of management and technical obstacles, while financial and external ones, though fewer, are also important for project success.

**Fig. 9:**
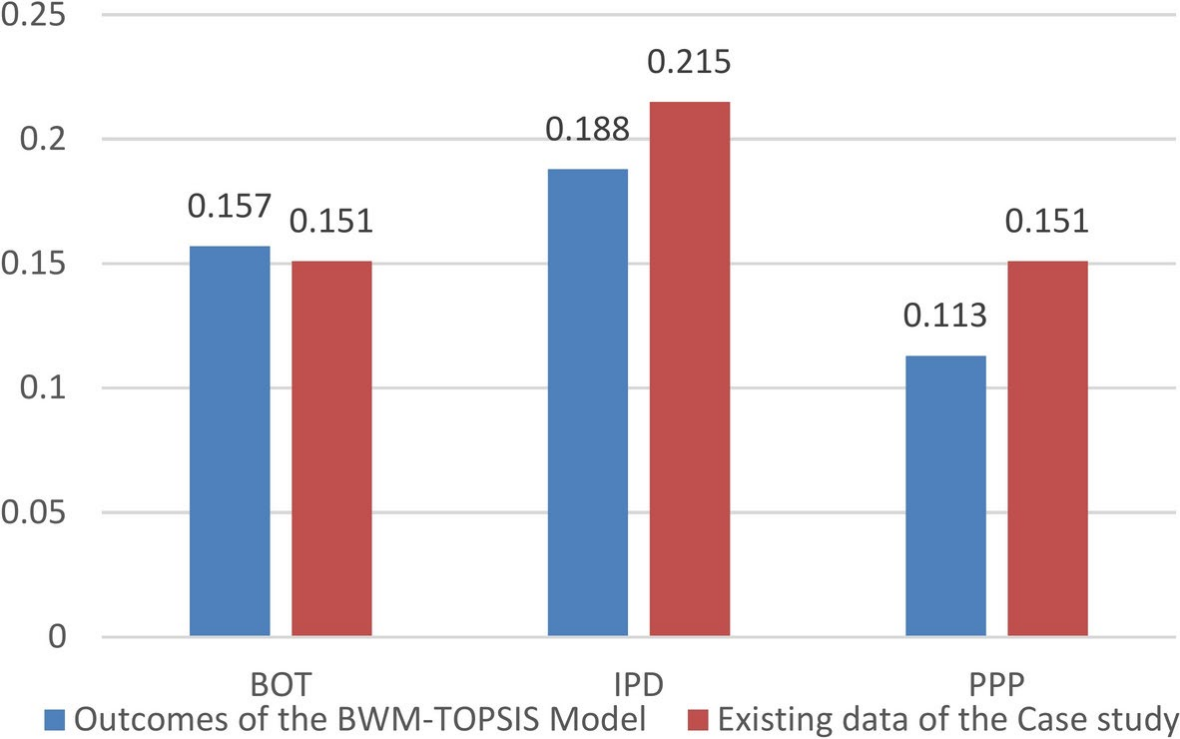
Comparison between results from existing data of the case study and outcomes of the BWM–TOPSIS model for methods considering all project phases.

**Fig. 10:**
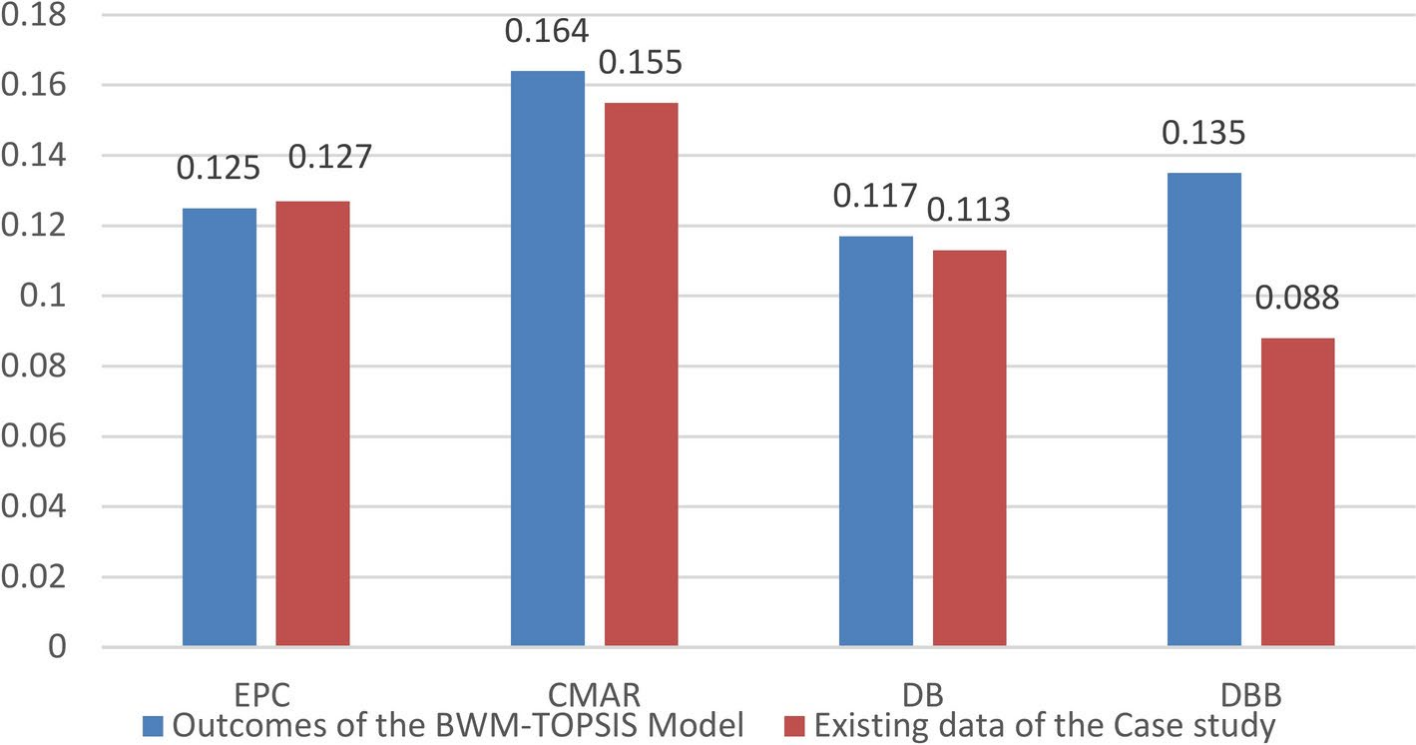
Comparison between results from existing data of the case study and outcomes of the BWM–TOPSIS model for methods considering development and construction phases.

The outcomes of applying the BWM–TOPSIS model and applying the suggested hybrid BWM–TOPSIS to the selected case study are summarized in Table 4 graphically presented in Figs. 9 and 10.

**Table 4:** Comparison between results from existing data of the case study and outcomes of the BWM–TOPSIS model.

	Existing data of the case study 1		Outcomes of the BWM–TOPSIS model		Relative error (All phases)	%
	Average normalized Dj (all phases)	Rank	Average normalized Dj (all phases)	Overall ranking		
EPC	0.127	5	0.125	5	0.016	1.60
CMAR	0.155	2	0.164	2	0.055	5.49
DB	0.113	6	0.117	6	0.034	3.42
DBB	0.088	7	0.135	4	0.348	34.81
BOT	0.151	3	0.157	3	0.038	3.82
IPD	0.215	1	0.188	1	0.144	14.36
PPP	0.151	3	0.113	7	0.336	33.63

Due to variations in projects’ conditions and constraints, the amount and types of risks in each risk category, evaluated using the Best Worst Method, the produced model, and case study analysis, have different results. Each project has unique qualities and conditions, thus different risk profiles. Variation in risk weights for each category affects the normalized Dj for TOPSIS-derived Project Delivery Methods (PDMs). Since each project’s risks are different, the PDM values will also change, underscoring the need to choose the best delivery mechanism for each project. Thus, due to value differences, individual risk evaluations are needed to pick PDMs accurately and effectively.

EPC, CMAR, DB, BOT, and IPD Project Delivery Methods (PDMs) show strong alignment with relative errors of less than 15% between theoretical studies and actual outcomes. The theoretical models closely match the amount and types of hazards in the data, explaining this tiny inaccuracy. Early stakeholder participation in IPD and contractor responsibility in EPC and CMAR assist in identifying and resolving issues before they worsen. Effective planning and execution, especially in DB and BOT approaches, analyze project timeframes, budgets, and technical needs to reduce execution variations. CMAR and BOT’s flexibility allows projects to react to unexpected events, reducing the error margin. IPD and CMAR’s collaborative techniques encourage early problem-solving. Resource management in EPC and DB improves operations and reduces costs.

The amount and types of risks revealed in case study data generally explain the significant inaccuracy between theoretical and practical studies in Project Delivery Methods (PDMs) like PPP and DBB. DBB projects have technical and schedule risks owing to the separation of design and construction, whereas PPP projects have financial and political risks due to their long-term public–private partnerships. The practical scenario’s PPP and DBB values differ from the theoretical one if the case study identifies more risks or if certain risks (like political instability or environmental conditions) are more impactful due to project circumstances. This risk-based divergence in data generates a relative inaccuracy, affecting PDM ranking and selection.

The high-ranked approach in this study was IPD, which indicates the importance of collaboration and stakeholder integration, which are vital in managing the complex offshore environment. The investigation into how the selected PDM influences the total risk profile empowers a manager to make necessary decisions, thus minimizing potential risks and enhancing the probability of project success.

This study’s outcome can benefit project managers and practitioners, as it provides a systematic methodology for obtaining the optimum PDM selected for the offshore construction project based on comprehensive risk analysis. The integrated BWM–TOPSIS model has allowed for the ranking of PDMs according to their capabilities of minimizing risks throughout the project’s entire life cycle.

The developed hybrid BWM–TOPSIS model can be applied effectively when certain critical factors are considered during project initiation, these factors are as follows:

1. Conduct an extensive identification of project risks across the entire project lifecycle, ensuring that potential risks are systematically identified at every stage.
2. Apply the BWM to calculate the weights of identified risks based on their significance, enabling a clear and accurate interpretation of the diverse risks associated with offshore projects.
3. Integrate the weights derived from BWM into the TOPSIS technique to rank project delivery methods (PDMs) according to their proximity to ideal solutions.
4. Evaluate the effectiveness of the highest-ranked PDM by analyzing its impact on the overall project risk assessment to confirm that the selected PDM adequately mitigates identified risks.
5. Establish a framework for continuous monitoring to evaluate the model’s performance and adapt it dynamically to changing project circumstances.

All these elements enable a methodical and efficient decision-making, risk management, and prioritization process for project delivery management in the offshore construction sector. Examples of worldwide projects utilizing the IPD method include the Johan Sverdrup Field in the North Sea, which is estimated to hold approximately 2.7 billion barrels of oil equivalents, marking it as one of the most significant discoveries on the Norwegian continental shelf in recent decades, with an expected operational lifespan exceeding 50 years. The Johan Sverdrup Project primarily employed the Engineering, Procurement, and Construction (EPC) method, noted for its integrated project management, efficient execution, and risk mitigation strategies. While elements characteristic of Integrated Project Delivery (IPD) are incorporated, contributing to enhanced project efficiency and success, the predominant framework remains EPC. This project also integrated advanced technologies, such as digital twinning and reservoir optimization, utilizing data analytics to improve operational efficiency and minimize environmental impact^9^.

Halliburton’s contract with Kuwait Oil Company (KOC) for implementing digital transformation solutions across Kuwait’s oil fields is based on the IPD approach. This initiative includes deploying automated work processes to optimize operational efficiency and increase production.

## Conclusions

The Best Worst Method (BWM) and TOPSIS are employed to determine the best project delivery strategy for each step of an offshore project’s lifecycle. The study presented the Risk Breakdown Structure (RBS) and a BWM–TOPSIS technique for choosing the most suitable delivery method for offshore projects. A comprehensive literature assessment is carried out to identify risks associated with various project delivery approach and offshore project life cycle.

Delphi techniques, combining qualitative and quantitative assessments, were used to identify critical risks and their impact on project objectives, with mitigation strategies proposed accordingly. The Risk Breakdown Structure categorized risks into Management, Technical, External & Site Conditions, and Financial & Economical hazards. Management risks were 52%, technical hazards 24%, and finance risks were more dispersed across all project delivery techniques except IPD. Weather, government laws, and improper waste management were the highest environmental risks. In two rounds of expert surveys, Primavera risk analysis was used to assess risks. The significant risks were weather, regulatory delays, money, project schedule mistakes, and managing waste. A second set of questions evaluated risks across stages using the Best–Worst Method (BWM). The significant development risks were design errors, economic policy changes, and inadequate feasibility studies. Site knowledge and financing were construction risks. Throughout operations, safety and the environment were important.

The hybrid BWM–TOPSIS model was subsequently created to choose the best PDM for offshore projects by comparing risk weights. TOPSIS ranked PDMs and found that Integrated Project Delivery (IPD) and Construction Manager at Risk (CMAR) were best for cooperation and risk management. Third was Build Operate Transfer (BOT), followed by inefficient Design Bid Build (DBB). IPD stakeholder integration enhanced risk management, cost predictability, and efficiency in construction. Two case studies validated the methodology, confirming IPD as the top PDM, with project-specific risks affecting other PDM rankings.

Analyzing normalized deviations to verify the model and show that real data matches theoretical conclusions, EPC, BOT, IPD, and CMAR balanced quality, integration, and flexibility. Due to their complexity, offshore projects must prioritize these objectives over cost and time. In conclusion, the BWM–TOPSIS model helps offshore decision-makers choose PDMs that mitigate project risks and optimize resource efficiency and success.

Early collaboration among all stakeholders in integrated project delivery (IPD) significantly enhances project outcomes by facilitating the early assessment of risks, resource optimization, allocation, and improving decision-making processes during the development phase. During the construction phase, where financial risks are highest, IPD’s collaborative nature involving all stakeholders from the outset improves risk management, cost predictability, and efficiency, thereby reducing the likelihood of delays and cost overruns. IPD ensures environmental effects are considered at every stage, resulting in improved resource efficiency, decreased waste, and minimal ecological disruption. Implementing IPD minimizes risks associated with improper operating techniques by establishing and consistently following the most efficient operating procedures. All these factors make IPD the best alternative for delivering offshore projects. DBB was deemed inefficient due to its limited collaboration and sequential structure, which often led to delays and cost overruns. EPC has been widely used for managing complex offshore projects due to its integrated approach. Design-Build (DB) is popular for its efficiency in combining design and construction. BOT and CMAR solve different difficulties in varied geographical environments, addressing the global offshore project market’s needs. For offshore projects where installation is critical, EPCI adds installation services to the EPC and lets a single contractor oversee all stages.

Due to political and logistical instability, Iraq and Iran need strict security. Without local knowledge, local content laws restrict employment and procurement. EPC is the best option since its integrated approach reduces risks and enhances production. Project delivery approaches that cover all stages, permit smooth transitions, and assess risk are the best. Development and construction approaches may focus on immediate outputs to satisfy project timelines and prices, whereas IPD prioritizes long-term value and sustainability. This restricted focus may favor solutions that improve initial project phases but not essential operational difficulties, increasing lifespan costs and performance issues after construction.

Egyptian offshore projects rarely adopt IPD due to cultural resistance, limited awareness of its benefits, and challenges in adapting it to local norms. Instead, they prefer traditional project delivery. Early stakeholder engagement in the IPD framework increases risk assessment, resource optimization, and decision-making, improving project results. In IPD, risk-sharing and reward encourage cooperation toward common goals, improving efficiency. IPD encourages collaboration between disciplines to discover and address design concerns. By including all stakeholders early on, IPD may reduce financial risks, especially during construction, and improve risk management, cost predictability, and operational efficiency. Continuous communication and problem-solving reduce mistakes and rework in this integrated method. IPD addresses environmental consequences throughout operation to improve resource efficiency, waste minimization, and ecological disturbance. Risk-sharing improves worker safety and decreases project accidents. IPD reduces the risk of improper operating processes by identifying and implementing efficient standards. Egyptian offshore projects benefit from IPD in terms of teamwork, efficiency, safety, design defects, operational procedures, and environmental impact. This holistic strategy improves project outcomes, making IPD a viable offshore development method in Egypt. The BWM–TOPSIS analysis found that offshore sector decision-makers must adopt a project delivery strategy that meets project requirements and manages risks throughout the project. Offshore building is difficult. Thus, this technique enhances project success, resource allocation, and results.

## Limitations and recommendations

Based on the process of conducting a risk assessment for the lifecycle of offshore projects through the development of a hybrid Best Worst Method (BWM) and Technique for Order Preference by Similarity to Ideal Solution (TOPSIS) model to identify the most suitable project delivery method, several recommendations for improvement are proposed:

1. This study relies on self-reported data from interviews and questionnaires, which may be subject to various biases. Respondents might underreport the most severe impacts of risks associated with different categories throughout the offshore project lifecycle. Future research could mitigate this limitation by incorporating additional project objectives, such as project performance metrics gathered from extensive corporate datasets.
2. The risk assessment and evaluation conducted in this study are primarily qualitative in nature. Future investigations could quantitatively analyze the time and cost impacts associated with the proposed delivery methods, thereby providing further insights and recommendations for stakeholders.
3. Future studies may explore applying alternative Multiple-Criteria Decision-Making (MCDM) approaches to enhance decision-making processes, such as integrating Fuzzy techniques.

## Data Availability

This manuscript does not report data generation or analysis.
